# Maternal alcohol consumption during pregnancy and child’s cognitive performance at 6–8 years of age in rural Burkina Faso: an observational study

**DOI:** 10.7717/peerj.3507

**Published:** 2017-06-30

**Authors:** Anselme Simeon Sanou, Abdoulaye Hama Diallo, Penny Holding, Victoria Nankabirwa, Ingunn Marie S. Engebretsen, Grace Ndeezi, James K. Tumwine, Nicolas Meda, Thorkild Tylleskar, Esperance Kashala-Abotnes

**Affiliations:** 1 Centre for International Health (CIH), Department of Global Public Health and Primary Health Care, Faculty of Medicine, University of Bergen, Bergen, Norway; 2 Department of Public Health, Centre MURAZ Research Institute, Ministry of Health, Bobo-Dioulasso, Burkina Faso; 3 Department of Public Health, University of Ouagadougou, Ouagadougou, Burkina Faso; 4 Saving Brains platform, Nairobi, Kenya; 5 Department of Epidemiology & Biostatistics, School of Public Health, Makerere University, Kampala, Uganda; 6 Centre for Intervention Science in Maternal and Child Health (CISMAC), Department of Global Public Health and Primary Health Care, Faculty of Medicine, University of Bergen, Bergen, Norway; 7 Department of Paediatrics and Child Health, Makerere University, Kampala, Uganda

**Keywords:** Maternal alcohol consumption, Cognitive test, Child development, Pregnancy, CCT-1, KABC-II, Children, Burkina Faso, Africa

## Abstract

**Background:**

In Burkina Faso, it is not uncommon for mothers to drink alcohol, even during pregnancy. We aimed to study the association between maternal alcohol consumption during pregnancy and the child’s cognitive performance using the Kaufman Assessment Battery for Children, 2nd edition (KABC-II) and the Children’s Category Test Level 1 (CCT-1) in rural Burkina Faso.

**Methods:**

We conducted a follow-up study of a community cluster-randomised Exclusive breastfeeding trial, and re-enrolled the children in rural Burkina Faso. A total of 518 children (268 boys and 250 girls) aged 6–8 years were assessed using the KABC-II and the CCT-1. We examined the effect size difference using Cohen’s d and conducted a linear regression analysis to examine the association.

**Results:**

Self-reported alcohol consumption during pregnancy was 18.5% (96/518). Children whose mothers reported alcohol consumption during pregnancy performed significantly poorly for memory and spatial abilities tests from small effect size difference for ‘Atlantis’ (0.27) and ‘Triangle’ (0.29) to moderate effect size difference for ‘Number recall’ (0.72) compared to children whose mothers did not consume alcohol during pregnancy; the exposed children scored significantly higher errors with a small effect size (0.37) at problem solving (CCT-1) test compared to unexposed children.

At unstandardized and standardized multivariable analysis, children whose mothers reported alcohol consumption during pregnancy performed significantly poorer for memory-‘Atlantis’ (*p* = 0.03) and ‘Number recall’ (*p* = 0.0001), and spatial ability tests-‘Triangle’ (*p* = 0.03); they scored significantly higher errors at problem solving CCT-1 test (*p* = 0.002); all the results were adjusted for age, sex, schooling, stunting, father’s education, mother’s employment and the promotion of exclusive breastfeeding. No statistical association was found for visual abilities-‘Conceptual Thinking’, ‘Face recognition’, ‘Story completion’, and reasoning tests-‘Rover’, ‘Block counting’, and ‘Pattern Reasoning’.

**Conclusion:**

Maternal alcohol consumption during pregnancy is associated with poorer cognitive performance for memory, spatial ability, and problem solving tests in the offspring in rural Burkina Faso. Futures studies needs to assess in more detail the maternal alcohol consumption patterns in Burkina Faso and possible preventive strategies.

## Introduction

The World Health Organization (WHO) recently stated that harmful consumption of alcohol is among the top five risk factors for disease, disability and death throughout the world. It is a causal factor in several diseases and injury conditions, and intake is on the increase, especially in low income countries (Rehm et al., 2009; WHO, 2014a).

Children exposed to prenatal alcohol have cognitive, physical and behavioural deficiencies (Popova et al., 2016b). Many studies have shown that regular and heavy consumption of alcohol during pregnancy are associated with neuropsychological and cognitive impairments in memory, executive function, processing speed, visual and spatial abilities, attention, language and academic achievement (Kodituwakku, Kalberg & May, 2001; O’Callaghan et al., 2007; Falgreen Eriksen et al., 2012; Flak et al., 2014). Recent reviews highlighted how prenatal alcohol can be sensitive on spatial abilities, reasoning (Mattson, Crocker & Nguyen, 2011), and memory (Du Plooy et al., 2016).

However, most of the evidence comes from high-income countries (Lewis et al., 2015; Lewis et al., 2016; Fan et al., 2016), and data are scarce in an African context where lack of resources, rural areas and home brewing alcohol consumption are common (Martinez et al., 2011). Burkina Faso is a country in Africa where the use of alcohol is increasing among women; it has among the highest national proportion of women consuming alcohol in the continent, 30% (Martinez et al., 2011). In 2016, a systematic review highlighted that the predicted prevalence of any amount of alcohol consumption during pregnancy among the general population in Burkina Faso was 11.3% (Popova et al., 2016a). According to the WHO, the level of total alcohol consumption was 6.8 litres of pure alcohol per capita for adults above 15 years of age from 2008 to 2010 (WHO, 2014b). The home brewed alcohol represented 84% of the type of alcohol consumed, followed by beer (10%), spirit (3%) and wine (3%) (WHO, 2014b).

Given the known harm from prenatal alcohol consumption and the evolving evidence of increasing drinking patterns among women in Africa, there is a need to explore alcohol consumption among pregnant women and its effect on the neuro-cognitive outcomes in their offspring in a context where lack of resources, rural areas and home brewing alcohol consumption are common. We aimed to study the association between maternal alcohol consumption during pregnancy and the offspring’s cognitive performance using the Kaufman Assessment Battery for Children, 2nd edition (KABC-II) and the Children’s Category Test Level 1 (CCT-1) in rural Burkina Faso.

## Subjects and Methods

### Study area, setting, study design and participants

Burkina Faso is a low income country located in the middle of West Africa; the population resides mainly in rural areas (70.1% in 2015), and the population aged 0–14 years was 46.3% in 2013 (INSD, 2016; UN Statistics, 2016). The literacy rate is very low and the mean years of education attained in women and girls was less than 3 years in 2013 (Patton et al., 2016). The official language in Burkina Faso is French. However, the country has more than 60 different ethnic groups. Several local languages are spoken in the study area Gouin, Karaboro, Dioula, Senoufo, Turka, Moore, and Fulfulde (Hama Diallo et al., 2012; Rossier et al., 2013; Ethnologue, 2016), which is a challenge when performing cognitive testing.

In 2006, a community-based cluster-randomised trial of children was conducted, the PROMISE Exclusive Breastfeeding (EBF) study. One of the sites was in rural Burkina Faso (Diallo et al., 2010; Diallo et al., 2011; Tylleskär et al., 2011; Hama Diallo et al., 2012). The sampling has been described (Diallo et al., 2010; Tylleskär et al., 2011). From 2013 to 2015, a cross-sectional follow-up study was conducted through the PROMISE Saving Brains study to assess the neuro-cognitive performance of the children aged 6–8 years old. We sought to re-enrol all children from the initial PROMISE EBF trial who were found to be alive and still residing in the study area.

### Outcome measures

The Kaufman Assessment Battery for Children, 2nd edition (KABC™-II) is an individually administered cognitive test with verbal and nonverbal components which has been used across diverse cultural contexts (Boivin et al., 1996; Ochieng, 2003; Kaufman & Kaufman, 2004; Malda et al., 2010). In Africa, it has been used to study cognitive development and nutrition in Ethiopia (Bogale et al., 2013), Democratic Republic of Congo (Boivin et al., 2013; Bumoko et al., 2015) and South Africa (Taljaard et al., 2013; Rochat et al., 2016), among HIV infected children in Uganda (Boivin et al., 2010; Ruel et al., 2012; Brahmbhatt et al., 2017), and cerebral malaria in Senegal (Boivin, 2002), and Uganda (Bangirana et al., 2009). KABC-II has different sub-tests and is used in children aged 3–18 years. The sub-tests (Appendix A) used in our study were:

• Atlantis: a measure of memory
• Conceptual Thinking: a measure of visual and spatial abilities
• Face recognition: a measure of visual and spatial abilities
• Story Completion: a measure of pattern recognition and reasoning
• Number Recall: a measure of memory
• Rover: a measure of spatial scanning and reasoning
• Triangle: a measure of spatial abilities and visualization
• Block Counting: a measure of reasoning
• Word Order: a measure of memory
• Pattern reasoning: a measure of reasoning and visualization (Kaufman & Kaufman, 2004; Bangirana et al., 2009).

The Children’s Category Test Level 1 (CCT-1) is a widely used non-verbal test developed to evaluate problem solving in children; it is fast and easy to administer (Boll, 1993; Hundal & Morris, 2011; Goudis, 2014). It was used to examine the effect of different exposures including health conditions like traumatic injuries (Moore, Donders & Thompson, 2004; Donders & Nesbit-Greene, 2004; Horneman & Emanuelson, 2009), brain dysfunction (Allen, Knatz & Mayfield, 2006; Bello, Allen & Mayfield, 2008), diseases (Rosenberg et al., 2010), marihuana and cocaine (Fried, Watkinson & Gray, 2005; Ga et al., 2015), disabilities (Hinton et al., 2004), chemical products (Debes et al., 2006; Wright et al., 2006; Jurewicz, Polańska & Hanke, 2013), and alcohol (Mattson et al., 1998). CCT-1 is an individually administered standardized test for children from 5 to 8 years to test their ability to solve problems on the basis of corrective feedback. It is presented in booklet form and consists of five subtests. At the end of the test, the total number of errors is counted. Children with more errors are the one who performed worst (Boll, 1993; Moore, Donders & Thompson, 2004; Allen, Knatz & Mayfield, 2006).

The KABC-II and the CCT-1 were administered by a team of four trained psychologists who spoke the local languages. The children were randomly assigned to the psychologists for assessment. The assessors administered individually the KABC-II and the CCT-1 during a one-to-one session. The instructions of the measures were translated in the main local language (Dioula) commonly spoken in the study area. Independent back translations were done.

### Exposure measure

Maternal alcohol consumption during pregnancy was the main exposure for this analysis. Information about maternal alcohol consumption during pregnancy was collected in a household interview with the caretaker prior to the neuro-cognitive assessment. Data collectors approached each child’s household to administer a questionnaire to the child’s caregiver during a one-to-one interview. Mothers were the primary respondents. A yes/no question of any alcohol consumption during pregnancy was asked. Of all the 554 caretakers, 518 were able to provide information on this question and 36 (6.5%) were not.

### Covariates

In the interview, questions were asked about background characteristics and socio-economic status that may influence the child’s performance. These include the child’s age, child’s schooling, father’s employment, father’s education (dichotomized to educated = at least one year in school, or not educated), mother’s age, mothers’ employment, mother’s education, current maternal alcohol status (a yes/no question of any current alcohol consumption), mother’s depression status using the Hopkins symptom checklist (Sirpal et al., 2016) (dichotomized to depression = at least one symptom in the checklist, no depression = no symptom in any of the checklist), mother’s chewing tobacco status (a yes/no question of current tobacco chewing), and presence of latrine in the compound (a yes/no question). Questions regarding past hospitalizations since birth of the child were asked and anthropometric data (height, age) were measured according to standard procedures (CDC, 2007) by a paediatrician at the study site. Stunting was defined as below-2 standard deviations of height-for-age.

Before the starting of data collection, field-testing and piloting of all the instruments was conducted to calibrate and standardize the assessment of cognitive measures and the data collection. For instance, the stadiometer for height was calibrated according to the instruction of manual, and the psychologists underwent field training and refresher training to standardize the way to administer the KABC-II and CCT-1 on local children prior to the study participants.

### Statistical analysis

Statistical analyses were conducted in several stages:

1. To examine within population variance of the sub-tests, the distribution of scores (mean, standard deviation, median, minimum and maximum) were used. Box-and-whisker plots per exposed and unexposed groups were used to illustrate the children’s performances on different sub-tests of KABC-II and CCT-1. Extreme scores were winsorized to discount the influence of outliers by replacing their values with the nearest scores within this range.
2. To examine the reliability of items of the sub-tests, split-half reliability coefficients were calculated for KABC-II (Kaufman & Kaufman, 2004; Malda et al., 2010) and Cronbach’s alpha coefficient was calculated for CCT-1 (Boll, 1993; Moore, Donders & Thompson, 2004; Allen, Knatz & Mayfield, 2006). The level of significance of the reliability coefficient was ≥0.7.
3. To examine the association between maternal alcohol consumption during pregnancy and cognitive performance of KABC-II and CCT-1, effect size differences using the Cohen’s d (Sullivan & Feinn, 2012; Cumming, 2014), and linear regression analysis were conducted. No validated norms of the KABC-II and the CCT-1 were available in Burkina Faso at the time of the study; we then used the raw scores instead of the scaled scores. However, all scores were standardized (*Z*) and analysis were conducted on both unstandardized and standardized scores. All the coefficients were adjusted for potential confounders including child’s age, sex, schooling, stunting, father’s employment, father’s education (Martinez et al., 2011; Falgreen Eriksen et al., 2012; Flak et al., 2014; Kesmodel et al., 2015) and the promotion of exclusive breastfeeding (‘intervention arm’ of the initial trial). A bivariate analysis between each covariate and the outcome was conducted (Table A1). STATA 13 was used to perform the analysis.

### Ethical considerations

Written informed consent was obtained from all care-takers in the study and oral assent was obtained from the children. The study was approved by the Institutional Review Board of Centre MURAZ number 008-2013/CE-CM.

**Figure 1:**
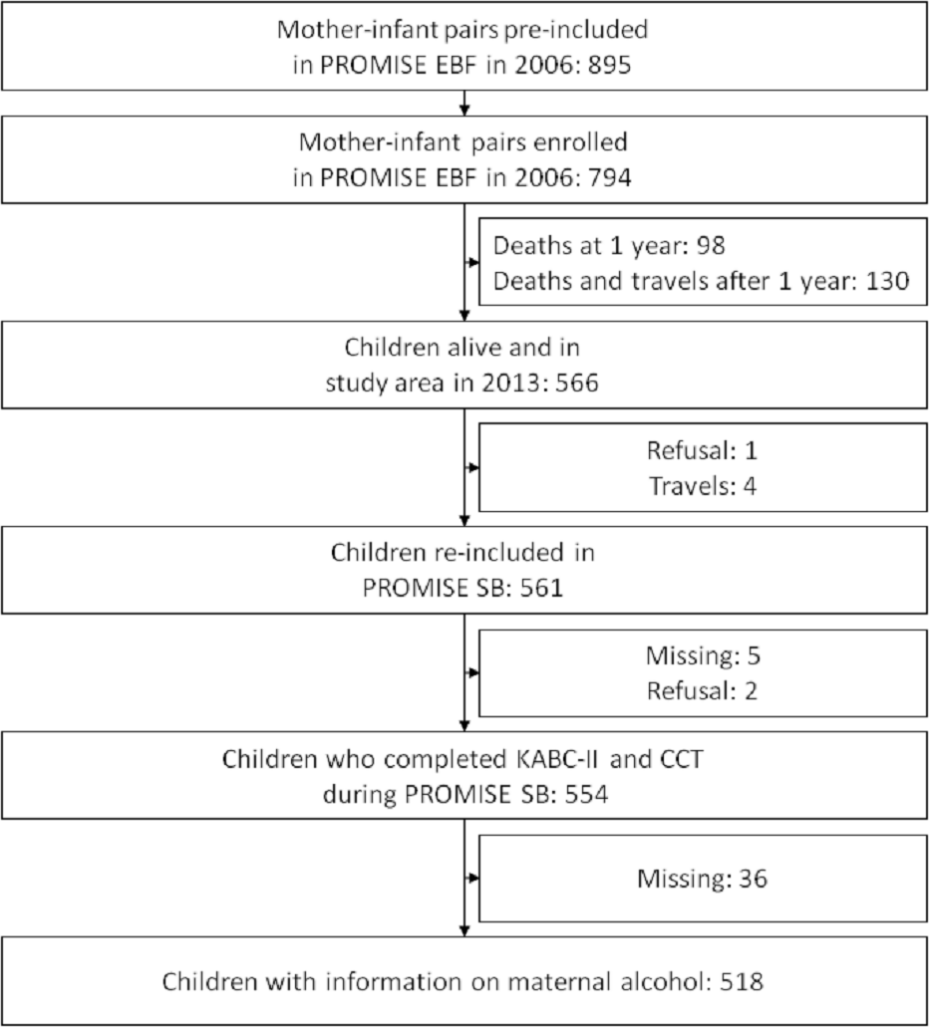
Study profile of children who completed the KABC-II and having information on maternal alcohol consumption during pregnancy at the PROMISE Saving Brains study in rural Burkina Faso.

## Results

### Study population

Of the initial 794 enrolled children in the PROMISE EBF trial in Burkina Faso site, 561 were alive, traced and re-consented for the follow-up study, 554 children completed the KABC-II and the CCT-1, and 518 children had information on their maternal alcohol consumption status (Fig. 1).

Of these, 51.7% (268/518) were boys, and 49.4% (256/518) were at school. The mean (± standard deviation, SD) age at assessment was 7.2 (±0.4 years), the median (interquartile range, IQR) was 7.2 (6.9–7.4) years and the range was 6.3–8 years. Of the mothers, 18.5% (96/518) reported to have consumed alcohol during the pregnancy and none of them had been more than 1 year in school. The mean (±SD) age of the mothers at assessment was 33.4 (±6.3 years). Of the fathers, 30.6% (156/510) had attended at least 1 year in school and 12.9% (67/518) had an employment. Three quarters of the compounds reported having a pit latrine 73.4%, (380/518) (Table 1).

**Table 1:** Description of the children who completed the KABC-II and CCT-1 from the PROMISE Saving Brains study in rural Burkina Faso.

	Total *N* = 518*N* (% )	Maternal alcohol *N* = 96(18.5)*N* (% )	No maternal alcohol *N* = 422(81.5)*N* (%)	*p*-value
Child age Mean ± SD (in years)	7.2 ± 0.4	7.2 ± 0.3	7.2 ± 0.4	0.38
Mothers age Mean ± SD (in years)	33.4 ± 6.3	34.4 ± 6.6	33.2 ± 6.2	0.17
Sex				0.7
Girls	250 (48.6)	45 (46.9)	205 (48.6)	
Boys	268 (51.4)	51 (53.1)	217 (51.4)	
Child in school				0.7
Yes	256 (49.4)	46 (47.9)	210 (49.8)	
No	262 (50.6)	50 (52.1)	212 (50.2)	
Stunting (<-2 SD in height-for-age)				0.8
No	426 (84.2)	79 (85.0)	347 (84.0)	
Yes	80 (15.8)	14 (15.0)	66 (16.0)	
Child has been hospitalized				0.6
No	391 (77.9)	71 (76.3)	320 (78.2)	
Yes	111 (22.1)	22 (23.7)	89 (21.8)	
Father employed				0.1
Yes	67 (12.9)	8 (8.3)	59 (14.0)	
No	451 (87.1)	88 (91.7)	363 (86.0)	
Father educated				0.8
Yes	156 (30.6)	28 (29.8)	128 (30.8)	
No	354 (69.4)	66 (70.2)	288 (69.2)	
Mother employed				0.1
Yes	26 (5.0)	2 (2.1)	24 (5.7)	
No	492 (95.0)	94 (97.9)	398 (94.3)	
Mother’s current alcohol consumption				0.0001
No	89 (17.2)	25 (26.3)	19 (4.5)	
Yes	428 (82.8)	70 (73.7)	403 (95.5)	
Mothers depression status				0.2
No	267 (51.5)	55 (57.29)	212 (50.2)	
Yes	251 (48.9)	41 (42.71)	210 (49.8)	
Mothers chewing tobacco				0.0001
No	495 (95.6)	85 (88.5)	410 (97.2)	
Yes	23 (4.4)	11 (11.5)	12 (2.8)	
Latrine in compound				0.003
Yes	380 (73.4)	59 (61.5)	321 (76.1)	
No	138 (26.6)	37 (38.5)	101 (23.9)	
PROMISE EBF intervention				0.07
Control arm	274 (52.9)	43 (44.8)	231 (54.7)	
Intervention arm	244 (47.1)	53 (55.2)	191 (45.3)	

**Notes.**

SD, Standard deviation

**Figure 2:**
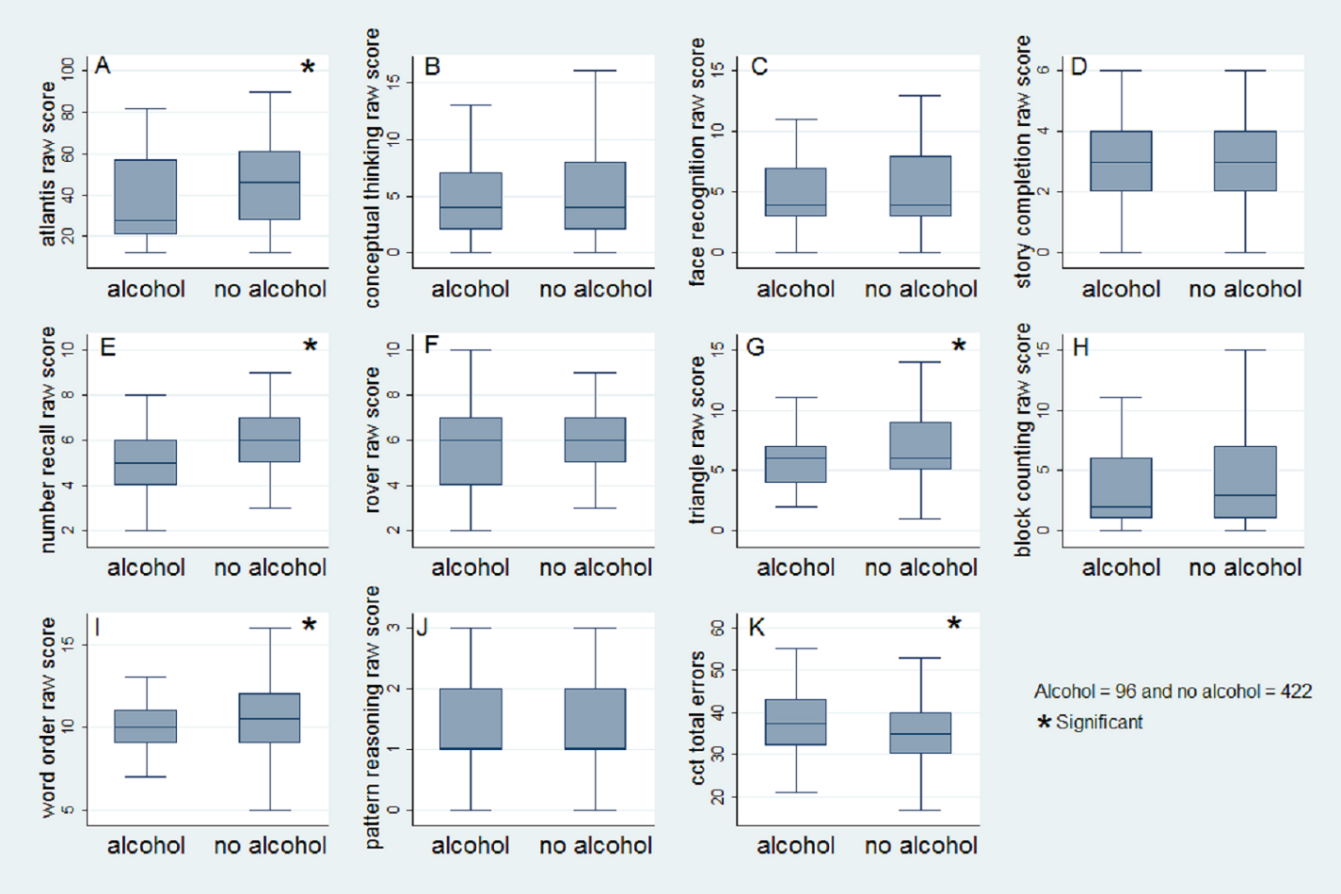
Box-and-whisker plots with median, interquartile range (box), minimum and maximum (whiskers) of child’s performance at KABC-II subtests and CCT-1 test by maternal alcohol consumption during pregnancy from the PROMISE Saving Brains study in rural Burkina Faso. (A) Atlantis raw score; (B) Conceptual thinking raw score; (C) Face recognition raw score; (D) Story completion raw score; (E) Number recall raw score; (F) Rover raw score; (G) Triangle raw score; (H) Block counting raw score; (I) Word order raw score; (J) Pattern reasoning raw score; (K) CCT-1 total errors.

**Table 2:** Tests description and internal consistency of 518 children who completed the KABC-II and CCT-1 from the PROMISE Saving Brains study in rural Burkina Faso.

Tests	Mean ± SD	Median (IQR)	Min score	Max score	Reliability coefficient
Atlantis	43.5 ± 19.4	43 (28–59)	12	90	0.96
Conceptual Thinking	5.1 ± 3.4	4 (2–8)	0	16	0.80
Face recognition	5.0 ± 3.0	4 (3–8)	0	13	0.74
Story completion	3.1 ± 1.3	3 (2–4)	0	6	0.44
Number recall	5.9 ± 1.8	6 (5–7)	2	9	0.76
Rover	6.0 ± 1.9	6 (5–7)	2	10	0.45
Triangle	6.7 ± 2.8	6 (5–8)	1	14	0.78
Block counting	4.1 ± 3.6	3 (1–7)	0	15	0.73
Word order	10.4 ± 1.8	10 (9–12)	5	16	0.64
Pattern reasoning	1.5 ± 1.0	1 (1–2)	0	3	0.56
CCT-1 errors	35.6 ± 7.2	35 (31–40)	17	55	0.82

**Notes.**

SD Standard deviation IQR Inter Quartile Range

On the KABC-II, sufficient variability (mean ± SD) of the raw scores was found for all the sub-tests except ‘Pattern Reasoning’ (Fig. 2 and Table 2). No child scored 0 in ‘Atlantis’, ‘Number recall’, ‘Rover’, ‘Triangle’ and ‘Word order’ (Fig. 2 and Table 2). The Split-half reliability coefficient was acceptable (>0.70) for all the sub-tests except ‘Story completion’, ‘Rover’, ‘Word order’ and ‘Pattern Reasoning’ (Table 2).

### Maternal alcohol consumption and cognitive performance

Children whose mothers reported alcohol consumption during pregnancy performed significantly poorly for memory and spatial abilities tests from small effect size difference for ‘Atlantis’ (0.27) and ‘Triangle’ (0.29) to moderate effect size difference for ‘Number recall’ (0.72) compared to children whose mothers did not consume alcohol during pregnancy; the exposed children scored significantly higher errors with a small effect size (0.37) at problem solving (CCT-1) test compared to unexposed children (Table 3).

At unstandardized and standardized multivariable analysis, children whose mothers reported alcohol consumption during pregnancy performed significantly poorer for memory-‘Atlantis’ (*p* = 0.03) and ‘Number recall’ (*p* = 0.0001), and spatial ability tests-‘Triangle’ (*p* = 0.03); they scored significantly higher errors at problem solving CCT-1 test (*p* = 0.002); all the results were adjusted for age, sex, schooling, stunting, father’s education, mother’s employment and the promotion of exclusive breastfeeding (Table 4). No statistical association was found for visual abilities-‘Conceptual Thinking’, ‘Face recognition’, ‘Story completion’, and reasoning tests-‘Rover’, ‘Block counting’, and ‘Pattern Reasoning’ (Table 4).

**Table 3:** Effect size and bivariate analysis between maternal alcohol consumption during pregnancy, KABC-II and CCT-1 performance of children from the PROMISE Saving Brains study in rural Burkina Faso.

	Effect size	Bivariate analysis		*p*-value
	Cohen’s d	Crude coefficient	95% CI	
Atlantis (memory)				
No alcohol		Reference		
Alcohol	0.27^a^	−5.45	−9.74 to −1.14	0.01
Conceptual Thinking (visual abilities)				
No alcohol		Reference		
Alcohol	0.02	−0.06	−0.82–0.69	0.86
Face recognition (visual abilities)				
No alcohol		Reference		
Alcohol	0.10	−0.28	−0.94–0.39	0.41
Story completion (reasoning)				
No alcohol		Reference		
Alcohol	0.05	−0.07	−0.3–0.2	0.62
Number recall (memory)				
No alcohol		Reference		
Alcohol	0.72^b^	−1.21	−1.59 to −0.84	<0.0001
Rover (reasoning)				
No alcohol		Reference		
Alcohol	0.11	−0.2	−0.6–0.2	0.29
Triangle (spatial abilities)				
No alcohol		Reference		
Alcohol	0.29^a^	−0.80	−1.42 to −0.18	0.01
Block counting (reasoning)				
No alcohol		Reference		
Alcohol	0.19	−0.71	−1.51–0.09	0.08
Word order (memory)				
No alcohol		Reference		
Alcohol	0.26	−0.5	−0.8 to −0.06	0.02
Pattern reasoning (reasoning)				
No alcohol		Reference		
Alcohol	0.09	−0.09	−0.3–0.1	0.42
CCT-1 errors (problem solving)				
No alcohol		Reference		
Alcohol	0.37^a^	2.7	1.1–4.3	0.001

**Notes.**

a Small effect size from 0.2 to 0.49.

b Moderate effect size from 0.5 to 0.79.

**Table 4:** Multivariable analysis between maternal alcohol consumption during pregnancy, KABC-II and CCT-1 performance of children from the PROMISE Saving Brains study in rural Burkina Faso.

	Unstandardized		Standardized	
	coefficient^a^ (95% CI)		coefficient^a^ (95% CI)	*p*-value
Atlantis (memory)				
No alcohol				
Alcohol	−4.4 (−8.6 to −0.3)		−0.2 (−0.4 to −0.01)	0.03
Conceptual Thinking (visual abilities)				
No alcohol				
Alcohol	−0.03 (−0.8–0.7)		−0.007 (−0.2–0.2)	0.9
Face recognition (visual abilities)				
No alcohol				
Alcohol	−0.1 (−0.8–0.5)		−0.04 (−0.3–0.2)	0.7
Story completion (reasoning)				
No alcohol				
Alcohol	−0.01 (−0.3–0.2)		−0.01 (−0.2–0.2)	0.9
Number recall (memory)				
No alcohol				
Alcohol	−1.1 (−1.5 to −0.7)		−0.6 (−0.8 to −0.4)	0.0001
Rover (reasoning)				
No alcohol				
Alcohol	−0.2 (−0.6–0.2)		−0.1 (−0.3–0.1)	0.3
Triangle (spatial abilities)				
No alcohol				
Alcohol	−0.6 (−1.2 to −0.03)		−0.2 (−0.4 to −0.01)	0.03
Block counting (reasoning)				
No alcohol				
Alcohol	−0.6 (−1.4 to −0.2)		−0.2 (−0.4 to −0.06)	0.1
Word order (memory)				
No alcohol				
Alcohol	−0.3 (−0.7–0.04)		−0.2 (−0.4–0.03)	0.08
Pattern Reasoning (reasoning)				
No alcohol				
Alcohol	−0.1 (−0.3–0.1)		−0.1 (−0.3–0.1)	0.3
CCT-1 errors (problem solving)				
No alcohol				
Alcohol	2.6 (0.9–4.2)		0.4 (0.1–0.6)	0.002

**Notes.**

a Adjusted for age, sex, schooling, stunting, father’s education, mother’s employment, and EBF (*N* = 498).

## Discussion

In the present study, we observed an association between maternal alcohol consumption in pregnancy and poorer cognitive performance for memory (‘Atlantis’ and ‘Number recall’), and spatial ability (‘Triangle’) tests as measured by the KABC-II and for problem solving as measured by CCT-1 among children aged 6 to 8 years in rural Burkina Faso. No statistical association was found for visual abilities (‘Conceptual Thinking’, ‘Face recognition’) and reasoning (‘Story completion’, ‘Rover’, ‘Block counting’, ‘Pattern Reasoning’).

Our study was conducted in an African rural context where home brewing is common and most commonly done by women. (Martinez et al., 2011; WHO, 2014b; Popova et al., 2016a). Its cost is low compared to commercially-made alcoholic beverages in many parts of Africa (Mccall, 1996; Willis, 2002) and quantifying its amount is challenging because home brews are often consumed in containers of various sizes (Hahn et al., 2012; Thakarar et al., 2016).

In its first application in rural Burkina Faso, we found variation in performances in the KABC-II and CCT-1. Children were positively engaged in carrying out the tests. Two things might explain the association between maternal alcohol consumption in pregnancy and poorer cognitive performance for ‘Atlantis’, ‘Number recall’, ‘Triangle’ and CCT-1. The first is the heavy home brewing consumption of alcohol during pregnancy. Numerous biological mechanisms have been suggested as contributing to alcohol-induced foetal damage, particularly deficits in brain function (Goodlett & Horn, 2001; Kim et al., 2016). The second is the good level of reliability for ‘Atlantis’, ‘Number recall’, ‘Triangle’ and ‘Block counting’ in accordance with the reliabilities reported in the KABC-II manual (Kaufman et al., 2005). Malda found similar results in India (Malda et al., 2010). These findings compare well some studies. In a recent systematic reviews of the literature which includes 33 relevant studies using cognitive test scores, children prenatally exposed to alcohol performed worse on problem solving, visual-spatial ability and specific domains of memory such as immediate or delayed recall memory compared to children who were prenatally unexposed (Du Plooy et al., 2016). Another review highlighted that heavy prenatal alcohol exposure had adverse effect on spatial abilities (Mattson, Crocker & Nguyen, 2011).

In the present study, we found no statistical association between maternal alcohol consumption in pregnancy and poorer cognitive performance for ‘Conceptual Thinking’, ‘Face recognition’, ‘Story completion’, ‘Rover’, ‘Block counting’ and ‘Pattern Reasoning’. Diverse explanations are possible as to why the children were not responsive to these tests. The most plausible is that the amount of maternal alcohol consumption during pregnancy was not enough to be associated with visual abilities and reasoning tests. In our study, the level of alcohol was unknown and might have been very low to detect significance association. These results are similar to other studies which found no difference between low to moderate alcohol consumption during pregnancy and neuro-cognitive outcomes among children (O’Callaghan et al., 2007; Alati et al., 2008; Kelly et al., 2012; Falgreen Eriksen et al., 2012; Kesmodel et al., 2012). Also, the reliability coefficient was low for ‘Story completion’, ‘Rover’ and ‘Pattern Reasoning’ and we found cultural unfamiliarity of the items for ‘Conceptual Thinking’ and ‘Face Recognition’. For example, ‘Face Recognition’ uses mainly photographs of faces from white people to which most children in rural Burkina Faso have not been exposed. In a study in rural Kenya using KABC-I, ‘Face Recognition’ has been adapted by substituted the photographs with those of persons from their region to increase the validity and the reliability of the measures (Holding et al., 2004). Given the fact that our study was implemented in similar context, such adaptations may have contributed to increase the responsiveness of children in our context. The reason of the low internal for ‘Story completion’, ‘Rover’ and ‘Pattern Reasoning’ might be explained by the weak understanding of the items; these tests measure reasoning and the understanding of the items might have been complex for the children due to the cultural context. The potential effect of cultural inappropriateness decreasing the performance has been described in multiple studies (Greenfield, 1997; Malda & Van der Vijver, 2008). While cognitive constructs appear to be universal (Koziol et al., 2014), the cultural context influences the engagement of the test taker in the testing process, and thus, potentially the reliability and validity of tests (Malda & Van der Vijver, 2008). Adaptations of the tests may therefore be needed to ensure the responsiveness of a test to group differences (Holding et al., 2004; Alcock et al., 2008). Thus, these sub-tests may differentiate children in our context after adaptations.

Our study has several strengths. Firstly, the risk of selection bias is small; the participants were part of a community-based cluster-randomised trial of children (Diallo et al., 2010; Diallo et al., 2011; Tylleskär et al., 2011; Hama Diallo et al., 2012). In addition, only two participants declined to be tested in the study. Secondly, the assessment was based on a standardized measure of cognition for children which has been widely used in a number of countries, also in Africa (Boivin, 2002; Bangirana et al., 2009; Boivin et al., 2010; Ruel et al., 2012; Bogale et al., 2013; Taljaard et al., 2013; Bumoko et al., 2015; Rochat et al., 2016; Brahmbhatt et al., 2017; Ajayi et al., 2017). In addition, the assessments were performed by trained psychologists who were blinded to the main exposure (maternal alcohol consumption). Thirdly, adjustment for the potential confounders was done in the analysis.

However, the study also has some limitations. The assessment of alcohol consumption was self-reported based on a dichotomous response without further probing. Therefore, we have no information about the volume, frequency and concentration of alcohol consumed. Misclassification of maternal alcohol consumption, in particular under-reporting and recall bias due to the recall time and social desirability cannot be excluded. However, the relatively high self-reported frequency of prenatal alcohol consumption might indicate that the population is naïve to health system information on the harmful effects on alcohol consumption in pregnancy and provide answers with limited social desirability. Given the relatively high frequency of drinking one could also assume that only ‘visible’ drinking of a certain ‘magnitude’ is reported and ‘sips’; ‘low alcohol beverages’ and ‘ritual drinking’ is not counted as drinking. Another limitation is the lack of overall reliability and validity of the measures which were used for the first time in the country and were not normed in the settings.

We consider this paper to be important as it demonstrates an association between maternal alcohol consumption and the poor cognitive performance among children in Burkina Faso. The study highlights the need to raise awareness of the risks of maternal alcohol consumption on the offspring’s cognitive performance. Healthcare professionals may have an important role in advising the public on its potential consequences. Prevention initiatives need to be designed and advice on abstaining from drinking during pregnancy needs to be provided. Strategies of monitoring alcohol intake on women and children may be considered during antenatal and postnatal visits. The cognitive outcome measures needs to be validated in the local context and culturally adapted.

## Conclusions

Maternal alcohol consumption during pregnancy is associated with poorer cognitive performance for memory, spatial ability, and problem solving tests in the offspring in rural Burkina Faso. Futures studies needs to assess in more detail the maternal alcohol consumption patterns in Burkina Faso and possible preventive strategies.

##  Supplemental Information

10.7717/peerj.3507/supp-1 Data S1 Dataset

## Appendix A

Outcome measures (Kaufman & Kaufman, 2004; Kaufman et al., 2005; Bangirana et al., 2009).

KABC-II is used in children aged 3–18 years. It has different sub-tests which include:

• **Atlantis**: The examiner teaches the child nonsense names for fanciful pictures of fish, plants and shells. The child demonstrates learning by pointing to each picture (out of an array of pictures) when it is named. ‘Atlantis’ is a measure of associative memory, and forms part of the learning ability scale;
• **Conceptual Thinking**: The child is presented a set of four or five pictures and must select the picture that does not belong with the set. It measures visual and spatial abilities and forms part of the simultaneous processing scale;
• **Face recognition**: The child looks at a photograph of either one or two faces for 5 s and then chooses the correct face (or faces) shown in a different pose from the original photograph. It measures visual and spatial abilities and forms part of the simultaneous processing scale;
• **Story Completion**: The child is shown a row of pictures that tell a story, with some of the pictures missing. The child should complete the story by selecting the missing pictures from a selection in their correct locations. ‘Story completion’ measures pattern recognition, reasoning and forms part of the planning ability scale;
• **Number Recall**: The child repeats a series of numbers in the same sequence the examiner said them. It measures memory span and forms part of the sequential processing scale;
• **Rover**: The child moves a toy dog to a bone on a checkerboard-like grid that contains obstacles (rocks and weeds) and tries to find path that takes the fewest moves. ‘Rover’ is a measure of spatial scanning, general sequential or deductive reasoning, number skills and forms part of the simultaneous processing scale;
• **Triangle**: For most items, the child assembles several identical foam triangles (blue on one side, yellow on the other) to match a picture of an abstract design. For easier items, the child assembles a set of colorful plastic shapes to match a model constructed by the examiner or shown on the easel. ‘Triangle’ measures spatial abilities, visualization and forms part of the simultaneous processing scale;
• **Block Counting**: The child counts the exact number of blocks in various pictures of stacks of blocks. The stacks are configured such that one of more blocks is hidden or partially hidden from view. ‘Block counting’ measures reasoning and forms part of the simultaneous processing scale;
• **Word Order**: The child touches a series of silhouettes of common objects in the same order as the examiner has named the objects. It measures memory span and forms part of the sequential processing scale;
• **Pattern reasoning**: The child is shown a series of stimuli that form a logical, linear pattern, with one stimulus missing. The child completes the pattern by selecting the correct stimulus from an array of 4–6 options at the bottom of the page. ‘Pattern Reasoning’ measures inductive reasoning, visualization and forms part of the simultaneous processing scale (Kaufman & Kaufman, 2004; Kaufman et al., 2005; Bangirana et al., 2009).

## Appendix B

**Table A1:** Crude coefficient from linear regression between covariates and the KABC-II test performance of children from the PROMISE Saving Brains study in rural Burkina Faso.

	Atlantis	Conceptual Thinking	Face recognition	Story completion	Number recall	Rover	Triangle	Block counting	Word order	Pattern reasoning	CCT-1 errors
Age, *N*	518	518	518	518	518	518	518	518	518	518	518
Crude	6.1	0.5	−0.07	0.3	0.4	0.06	0.8	0.3	0.7	0.04	−0.9
95% CI	1.4–10.7	−0.3–1.3	−0.8–0.6	−0.004–0.6	−0.04–0.8	−0.4–0.5	0.1–1.4	−0.5–1.2	0.3–1.2	−0.2–0.3	−2.6 –0.8
*p*-value	0.01	0.2	0.8	0.05	0.08	0.7	0.02	0.4	0.001	0.7	0.3
Sex, *N*	518	518	518	518	518	518	518	518	518	518	518
Crude	2.13	0.5	0.09	−0.06	0.2	0.2	0.5	0.3	0.1	0.1	0.8
95% CI	−1.2–5.5	−0.1–1.1	−0.4–0.6	−0.3–1.7	−0.06–0.5	−0.1–0.5	0.04–1.0	−0.3–0.9	−0.1–0.5	−0.06–0.3	−0.4–2.0
*p*-value	0.2	0.1	0.7	0.6	0.1	0.1	0.03	0.3	0.3	0.2	0.2
Child in school, *N*	518	518	518	518	518	518	518	518	518	518	518
Crude	11.0	0.6	0.6	0.4	0.3	0.6	1.9	0.6	0.9	0.01	1.2
95% CI	7.8–14.2	0.03–1.2	0.07–1.1	0.1–0.6	0.04–0.6	0.3–0.9	1.5–2.4	0.01–1.2	0.6–1.3	−0.1–0.2	0.02–2.5
*p*-value	0.0001	0.03	0.02	0.001	0.02	0.0001	0.0001	0.04	0.0001	0.8	0.04
Stunting, *N*	506	506	506	506	506	506	506	506	506	506	506
Crude	8.1	0.8	0.6	0.06	0.3	0.4	1.06	0.6	0.6	−0.1	1.7
95% CI	3.5–12.7	0.1–1.7	−0.1–1.3	−0.3–0.4	−0.07–0.7	0.0006–0.9	0.4–1.7	−0.3–1.5	0.1–1.0	−0.4–0.09	0.01–3.5
*p*-value	0.001	0.03	0.09	0.7	0.1	0.05	0.002	0.2	0.006	0.2	0.04
Father educated, *N*	510	510	510	510	510	510	510	510	510	510	510
Crude	5.4	0.5	1.1	0.2	0.5	0.3	0.8	0.5	0.4	0.06	0.9
95% CI	1.8–9.0	−0.1–1.1	0.5–1.7	−0.08–0.4	0.1–0.8	−0.03–0.6	0.2–1.3	−0.2–1.2	0.1–0.8	−0.1–0.2	−0.3–2.3
*p*-value	0.004	0.1	0.0001	0.1	0.006	0.08	0.004	0.1	0.01	0.5	0.1
Mother’s employment, *N*	518	518	518	518	518	518	518	518	518	518	518
Crude	5.8	0.2	1.6	0.1	1.4	0.2	0.9	1.6	0.9	0.1	3.7
95% CI	−1.8–13.4	−1.0–1.6	0.4–2.7	−0.3–0.6	0.7–2.1	−0.5–0.9	−0.1–2.0	0.2–3.0	0.1–1.6	−0.3–0.5	0.8–6.6
*p*-value	0.1	0.6	0.008	0.5	0.0001	0.5	0.07	0.02	0.01	0.5	0.01
PROMISE EBF intervention, *N*	518	518	518	518	518	518	518	518	518	518	518
Crude	−0.8	−0.8	−0.3	−0.1	−0.3	−0.01	−0.4	0.06	−0.006	−0.06	1.2
95% CI	−4.2–2.5	−1.4 to −0.2	−0.8–0.2	−0.3–0.07	−0.6 to −0.001	−0.3–0.3	−0.9–0.04	−0.5–0.6	−0.3–0.3	−0.2–0.1	−0.04–2.4
*p*-value	0.6	0.005	0.2		0.04	0.9	0.07	0.8	0.9	0.4	0.059

## Additional Information and Declarations

### Data Availability

The following information was supplied regarding data availability: The raw data has been uploaded as Data S1.

## References

[ref-1] Ajayi OR, Matthews G, Taylor M, Kvalsvig J, Davidson LL, Kauchali S, Mellins CA (2017). Factors associated with the health and cognition of 6–8 year old children in KwaZulu-Natal, South Africa. Tropical Medicine & International Health.

[ref-2] Alati R, Macleod J, Hickman M, Sayal K, May M, Smith GD, Lawlor DA (2008). Intrauterine exposure to alcohol and tobacco use and childhood IQ: findings from a parental-offspring comparison within the avon longitudinal study of parents and children. Pediatric Research.

[ref-3] Alcock KJ, Holding PA, Mung’ala-Odera V, Newton CRJC (2008). Constructing tests of cognitive abilities for schooled and unschooled children. Journal of Cross-Cultural Psychology.

[ref-4] Allen DN, Knatz DT, Mayfield J (2006). Validity of the children’s category test-level 1 in a clinical sample with heterogeneous forms of brain dysfunction. Archives of Clinical Neuropsychology.

[ref-5] Bangirana P, Seggane-Musisi null, Allebeck P, Giordani B, John CC, Opoka OR, Byarugaba J, Ehnvall A, Boivin MJ (2009). A preliminary examination of the construct validity of the KABC-II in Ugandan children with a history of cerebral malaria. African Health Sciences.

[ref-6] Bello DT, Allen DN, Mayfield J (2008). Sensitivity of the children’s category test level 2 to brain dysfunction. Archives of Clinical Neuropsychology.

[ref-7] Bogale A, Stoecker BJ, Kennedy T, Hubbs-Tait L, Thomas D, Abebe Y, Hambidge KM (2013). Nutritional status and cognitive performance of mother-child pairs in Sidama, Southern Ethiopia. Maternal & Child Nutrition.

[ref-8] Boivin MJ (2002). Effects of early cerebral malaria on cognitive ability in Senegalese children. Journal of Developmental and Behavioral Pediatrics.

[ref-9] Boivin MJ, Chounramany C, Giordani B, Xaisida S, Choulamountry L (1996). Validating a cognitive ability testing protocol with Lao children for community development applications. Neuropsychology.

[ref-10] Boivin MJ, Okitundu D, Makila-Mabe Bumoko G, Sombo M-T, Mumba D, Tylleskar T, Page CF, Tamfumx Muyembe J-J, Tshala-Katumbay D (2013). Neuropsychological effects of konzo: a neuromotor disease associated with poorly processed cassava. Pediatrics.

[ref-11] Boivin MJ, Ruel TD, Boal HE, Bangirana P, Cao H, Eller LA, Charlebois E, Havlir DV, Kamya MR, Achan J, Akello C, Wong JK (2010). HIV-subtype A is associated with poorer neuropsychological performance compared with subtype D in antiretroviral therapy-naive Ugandan children. AIDS.

[ref-12] Boll TJ (1993). Manual for children’s category test.

[ref-13] Brahmbhatt H, Boivin M, Ssempijja V, Kagaayi J, Kigozi G, Serwadda D, Violari A, Gray RH (2017). Impact of HIV and atiretroviral therapy on neurocognitive outcomes among school aged children. Journal of Acquired Immune Deficiency Syndromes.

[ref-14] Bumoko GM-M, Sadiki NH, Rwatambuga A, Kayembe KP, Okitundu DL, Mumba Ngoyi D, Muyembe J-JT, Banea J-P, Boivin MJ, Tshala-Katumbay D (2015). Lower serum levels of selenium, copper, and zinc are related to neuromotor impairments in children with konzo. Journal of the Neurological Sciences.

[ref-15] CDC (2007). Anthropometry procedures manual. http://www.cdc.gov/nchs/data/nhanes/nhanes_07_08/manual_an.pdf.

[ref-16] Cumming G (2014). The new statistics: why and how. Psychological Science.

[ref-17] Debes F, Budtz-Jørgensen E, Weihe P, White RF, Grandjean P (2006). Impact of prenatal methylmercury exposure on neurobehavioral function at age 14 years. Neurotoxicology and Teratology.

[ref-18] Diallo AH, Meda N, Ouedraogo WT, Cousens S, Tylleskar T (2011). A prospective study on neonatal mortality and its predictors in a rural area in Burkina Faso: can MDG-4 be met by 2015?. Journal of Perinatology.

[ref-19] Diallo AH, Meda N, Zabsonré E, Sommerfelt H, Cousens S, Tylleskär T (2010). Perinatal mortality in rural Burkina Faso: a prospective community-based cohort study. BMC Pregnancy and Childbirth.

[ref-20] Donders J, Nesbit-Greene K (2004). Predictors of neuropsychological test performance after pediatric traumatic brain injury. Assessment.

[ref-21] Du Plooy CP, Malcolm-Smith S, Adnams CM, Stein DJ, Donald KA (2016). The effects of prenatal alcohol exposure on episodic memory functioning: a systematic review. Archives of Clinical Neuropsychology.

[ref-22] Ethnologue (2016). Languages of Burkina Faso. http://www.ethnologue.com/map/BF.

[ref-23] Falgreen Eriksen H-L, Mortensen EL, Kilburn T, Underbjerg M, Bertrand J, Støvring H, Wimberley T, Grove J, Kesmodel US (2012). The effects of low to moderate prenatal alcohol exposure in early pregnancy on IQ in 5-year-old children. BJOG.

[ref-24] Fan J, Jacobson SW, Taylor PA, Molteno CD, Dodge NC, Stanton ME, Jacobson JL, Meintjes EM (2016). White matter deficits mediate effects of prenatal alcohol exposure on cognitive development in childhood. Human Brain Mapping.

[ref-25] Flak AL, Su S, Bertrand J, Denny CH, Kesmodel US, Cogswell ME (2014). The association of mild, moderate, and binge prenatal alcohol exposure and child neuropsychological outcomes: a meta-analysis. Alcoholism, Clinical and Experimental Research.

[ref-26] Fried PA, Watkinson B, Gray R (2005). Neurocognitive consequences of marihuana–a comparison with pre-drug performance. Neurotoxicology and Teratology.

[ref-27] Ga R, L G, C L, Nl D (2015). Effects of prenatal cocaine exposure on adolescent development. Neurotoxicology and Teratology.

[ref-28] Goodlett CR, Horn KH (2001). Mechanisms of alcohol-induced damage to the developing nervous system. Alcohol Research & Health.

[ref-29] Goudis N (2014). Statistical properties and clinical utility of the children’s category test–Level 1. Dissertation/Thesis.

[ref-30] Greenfield PM (1997). You can’t take it with you: why ability assessments don’t cross cultures. American Psychologist.

[ref-31] Hahn JA, Dobkin LM, Mayanja B, Emenyonu NI, Kigozi IM, Shiboski S, Bangsberg DR, Gnann H, Weinmann W, Wurst FM (2012). Phosphatidylethanol (PEth) as a biomarker of alcohol consumption in HIV-positive patients in sub-Saharan Africa. Alcoholism, Clinical and Experimental Research.

[ref-32] Hama Diallo A, Meda N, Sommerfelt H, Traore GS, Cousens S, Tylleskar T, PROMISE-EBF Study Group (2012). The high burden of infant deaths in rural Burkina Faso: a prospective community-based cohort study. BMC Public Health.

[ref-33] Hinton VJ, De Vivo DC, Fee R, Goldstein E, Stern Y (2004). Investigation of poor academic achievement in children with duchenne muscular dystrophy. Learning Disabilities Research & Practice: A Publication of the Division for Learning Disabilities, Council for Exceptional Children.

[ref-34] Holding PA, Taylor HG, Kazungu SD, Mkala T, Gona J, Mwamuye B, Mbonani L, Stevenson J (2004). Assessing cognitive outcomes in a rural African population: development of a neuropsychological battery in Kilifi District, Kenya. Journal of the International Neuropsychological Society.

[ref-35] Horneman G, Emanuelson I (2009). Cognitive outcome in children and young adults who sustained severe and moderate traumatic brain injury 10 years earlier. Brain Injury.

[ref-36] Hundal JS, Morris J (2011). Clinical validity of the children’s category test-level 2 in a mixed sample of school-aged children. Archives of Clinical Neuropsychology.

[ref-37] INSD (2016). Chiffres clés de l’institut national de la statistique et de la demographie. http://www.insd.bf/n/.

[ref-38] Jurewicz J, Polańska K, Hanke W (2013). Chemical exposure early in life and the neurodevelopment of children–an overview of current epidemiological evidence. Annals of Agricultural and Environmental Medicine.

[ref-39] Kaufman AL, Kaufman NL (2004). Kaufman assessment battery for children manual.

[ref-40] Kaufman AS, Lichtenberger EO, Fletcher-Janzen E, Kaufman NL (2005). Essentials of KABC-II Assessment.

[ref-41] Kelly YJ, Sacker A, Gray R, Kelly J, Wolke D, Head J, Quigley MA (2012). Light drinking during pregnancy: still no increased risk for socioemotional difficulties or cognitive deficits at 5 years of age?. Journal of Epidemiology and Community Health.

[ref-42] Kesmodel US, Bertrand J, Støvring H, Skarpness B, Denny CH, Mortensen EL, Lifestyle During Pregnancy Study Group (2012). The effect of different alcohol drinking patterns in early to mid pregnancy on the child’s intelligence, attention, and executive function. An International Journal of Obstetrics and Gynaecology.

[ref-43] Kesmodel US, Kjaersgaard MIS, Denny CH, Bertrand J, Skogerbø Å, Eriksen H-LF, Bay B, Underbjerg M, Mortensen EL (2015). The association of pre-pregnancy alcohol drinking with child neuropsychological functioning. BJOG.

[ref-44] Kim YY, Roubal I, Lee YS, Kim JS, Hoang M, Mathiyakom N, Kim Y (2016). Alcohol-induced molecular dysregulation in human embryonic stem cell-derived neural precursor cells. PLOS ONE.

[ref-45] Kodituwakku PW, Kalberg W, May PA (2001). The effects of prenatal alcohol exposure on executive functioning. Alcohol Research & Health.

[ref-46] Koziol LF, Barker LA, Joyce AW, Hrin S (2014). Structure and function of large-scale brain systems. Applied Neuropsychology: Child.

[ref-47] Lewis CE, Thomas KGF, Dodge NC, Molteno CD, Meintjes EM, Jacobson JL, Jacobson SW (2015). Verbal learning and memory impairment in children with fetal alcohol spectrum disorders. Alcoholism, Clinical and Experimental Research.

[ref-48] Lewis CE, Thomas KGF, Molteno CD, Kliegel M, Meintjes EM, Jacobson JL, Jacobson SW (2016). Prospective memory impairment in children with prenatal alcohol exposure. Alcoholism, Clinical and Experimental Research.

[ref-49] Malda M, Van der Vijver FJR (2008). Adapting a cognitive test for a different culture: an illustration of qualitative procedures. Psychology Science Quarterly.

[ref-50] Malda M, Van de Vijver FJR, Srinivasan K, Transler C, Sukumar P (2010). Traveling with cognitive tests: testing the validity of a KABC-II adaptation in India. Assessment.

[ref-51] Martinez P, Røislien J, Naidoo N, Clausen T (2011). Alcohol abstinence and drinking among African women: data from the world health surveys. BMC Public Health.

[ref-52] Mattson SN, Crocker N, Nguyen TT (2011). Fetal alcohol spectrum disorders: neuropsychological and behavioral features. Neuropsychology Review.

[ref-53] Mattson SN, Riley EP, Gramling L, Delis DC, Jones KL (1998). Neuropsychological comparison of alcohol-exposed children with or without physical features of fetal alcohol syndrome. Neuropsychology.

[ref-54] Mccall M (1996). Rural brewing, exclusion, and development policy-making. Gender and Development.

[ref-55] Moore BA, Donders J, Thompson EH (2004). Validity of the children’s category test-level 1 after pediatric traumatic brain injury. Archives of Clinical Neuropsychology.

[ref-56] O’Callaghan FV, O’Callaghan M, Najman JM, Williams GM, Bor W (2007). Prenatal alcohol exposure and attention, learning and intellectual ability at 14 years: a prospective longitudinal study. Early Human Development.

[ref-57] Ochieng CO (2003). Meta-analysis of the validation studies of the kaufman assessment battery for children. International Journal of Testing.

[ref-58] Patton GC, Sawyer SM, Santelli JS, Ross DA, Afifi R, Allen NB, Arora M, Azzopardi P, Baldwin W, Bonell C, Kakuma R, Kennedy E, Mahon J, McGovern T, Mokdad AH, Patel V, Petroni S, Reavley N, Taiwo K, Waldfogel J, Wickremarathne D, Barroso C, Bhutta Z, Fatusi AO, Mattoo A, Diers J, Fang J, Ferguson J, Ssewamala F, Viner RM (2016). Our future: a Lancet commission on adolescent health and wellbeing. Lancet.

[ref-59] Popova S, Lange S, Probst C, Shield K, Kraicer-Melamed H, Ferreira-Borges C, Rehm J (2016a). Actual and predicted prevalence of alcohol consumption during pregnancy in the WHO African Region. Tropical Medicine & International Health.

[ref-60] Popova S, Lange S, Shield K, Mihic A, Chudley AE, Mukherjee RAS, Bekmuradov D, Rehm J (2016b). Comorbidity of fetal alcohol spectrum disorder: a systematic review and meta-analysis. Lancet.

[ref-61] Rehm J, Mathers C, Popova S, Thavorncharoensap M, Teerawattananon Y, Patra J (2009). Global burden of disease and injury and economic cost attributable to alcohol use and alcohol-use disorders. Lancet.

[ref-62] Rochat TJ, Houle B, Stein A, Coovadia H, Coutsoudis A, Desmond C, Newell M-L, Bland RM (2016). Exclusive breastfeeding and cognition, executive function, and behavioural disorders in primary school-aged children in rural South Africa: a cohort analysis. PLOS Medicine.

[ref-63] Rosenberg AA, Lee NR, Vaver KN, Werner D, Fashaw L, Hale K, Waas N (2010). School-age outcomes of newborns treated for persistent pulmonary hypertension. Journal of Perinatology.

[ref-64] Rossier J, Ouedraogo A, Dahourou D, Verardi S, Meyer de Stadelhofen F (2013). Personality and personality disorders in urban and rural Africa: results from a field trial in Burkina Faso. Frontiers in Psychology.

[ref-65] Ruel TD, Boivin MJ, Boal HE, Bangirana P, Charlebois E, Havlir DV, Rosenthal PJ, Dorsey G, Achan J, Akello C, Kamya MR, Wong JK (2012). Neurocognitive and motor deficits in HIV-infected Ugandan children with high CD4 cell counts. Clinical Infectious Diseases.

[ref-66] Sirpal MK, Haugen W, Sparle K, Haavet OR (2016). Validation study of HSCL-10, HSCL-6, WHO-5 and 3-key questions in 14–16 year ethnic minority adolescents. BMC Family Practice.

[ref-67] Sullivan GM, Feinn R (2012). Using effect size–or why the *P* value is not enough. Journal of Graduate Medical Education.

[ref-68] Taljaard C, Covic NM, Van Graan AE, Kruger HS, Smuts CM, Baumgartner J, Kvalsvig JD, Wright HH, Van Stuijvenberg ME, Jerling JC (2013). Effects of a multi-micronutrient-fortified beverage, with and without sugar, on growth and cognition in South African schoolchildren: a randomised, double-blind, controlled intervention. The British Journal of Nutrition.

[ref-69] Thakarar K, Asiimwe SB, Cheng DM, Forman L, Ngabirano C, Muyindike WR, Emenyonu NI, Samet JH, Hahn JA (2016). Alcohol consumption in ugandan hiv-infected household-brewers versus non-brewers. AIDS and Behavior.

[ref-70] Tylleskär T, Jackson D, Meda N, Engebretsen IMS, Chopra M, Diallo AH, Doherty T, Ekström E-C, Fadnes LT, Goga A, Kankasa C, Klungsøyr JI, Lombard C, Nankabirwa V, Nankunda JK, Van de Perre P, Sanders D, Shanmugam R, Sommerfelt H, Wamani H, Tumwine JK (2011). Exclusive breastfeeding promotion by peer counsellors in sub-Saharan Africa (PROMISE-EBF): a cluster-randomised trial. Lancet.

[ref-71] UN Statistics (2016). Profile of Burkina Faso, World statistics pocketbook. http://data.un.org/CountryProfile.aspx?crName=burkina%20faso.

[ref-72] WHO (2014a). Global status report on alcohol and health. http://www.who.int/substance_abuse/publications/global_alcohol_report/en/.

[ref-73] WHO (2014b). Global information system on alcohol and health. http://apps.who.int/gho/data/?showonly=GISAH&theme=main.

[ref-74] Willis J (2002). Potent brews: a social history of alcohol in East Africa 1850–1999.

[ref-75] Wright RO, Amarasiriwardena C, Woolf AD, Jim R, Bellinger DC (2006). Neuropsychological correlates of hair arsenic, manganese, and cadmium levels in school-age children residing near a hazardous waste site. NeuroToxicology.

